# Diagnostic accuracy of brain age prediction in a memory clinic population and comparison with clinically available volumetric measures

**DOI:** 10.1038/s41598-023-42354-0

**Published:** 2023-09-11

**Authors:** Karin Persson, Esten H. Leonardsen, Trine Holt Edwin, Anne-Brita Knapskog, Gro Gujord Tangen, Geir Selbæk, Thomas Wolfers, Lars T. Westlye, Knut Engedal

**Affiliations:** 1 The Norwegian National Centre for Ageing and Health, Vestfold Hospital Trust, Tønsberg, Norway; 2 Department of Geriatric Medicine, Oslo University Hospital, Oslo, Norway; 3 Department of Psychology, University of Oslo, Oslo, Norway; 4 Norwegian Centre for Mental Disorders Research (NORMENT), Oslo University Hospital and Institute of Clinical Medicine, University of Oslo, Oslo, Norway; 5 Faculty of Medicine, University of Oslo, Oslo, Norway; 6 Department of Psychiatry and Psychotherapy, Tübingen Center for Mental Health, University of Tübingen, Tübingen, Germany

**Keywords:** Biomarkers, Preclinical research, Translational research, Health care, Medical research

## Abstract

The aim of this study was to assess the diagnostic validity of a deep learning-based method estimating brain age based on magnetic resonance imaging (MRI) and to compare it with volumetrics obtained using NeuroQuant (NQ) in a clinical cohort. Brain age prediction was performed on minimally processed MRI data using deep convolutional neural networks and an independent training set. The brain age gap (difference between chronological and biological age) was calculated, and volumetrics were performed in 110 patients with dementia (Alzheimer’s disease, frontotemporal dementia (FTD), and dementia with Lewy bodies), and 122 with non-dementia (subjective and mild cognitive impairment). Area-under-the-curve (AUC) based on receiver operating characteristics and logistic regression analyses were performed. The mean age was 67.1 (9.5) years and 48.7% (113) were females. The dementia versus non-dementia sensitivity and specificity of the volumetric measures exceeded 80% and yielded higher AUCs compared to BAG. The explained variance of the prediction of diagnostic stage increased when BAG was added to the volumetrics. Further, BAG separated patients with FTD from other dementia etiologies with > 80% sensitivity and specificity. NQ volumetrics outperformed BAG in terms of diagnostic discriminatory power but the two methods provided complementary information, and BAG discriminated FTD from other dementia etiologies.

## Introduction

The number of patients suffering from dementia is rapidly increasing worldwide as populations grow older. To date no curative treatment is available for any dementia disorder^1^. However, new treatment possibilities targeted at Alzheimer’s disease (AD) are evolving, prompting the need for tools to aid early diagnosis and predict future cognitive decline^2^. This is of utmost importance as potentially disease-modifying treatment strategies will target early pathophysiological changes.

The use of artificial intelligence (AI) to improve and facilitate early diagnosis, planning, and follow-up of treatment has emerged rapidly in many medical fields, including the field of cognitive impairment and dementia^3^. A recent report published by the Norwegian directorate of health encouraged increased use of AI in radiology to improve early diagnosis and provide decision support^4^.

AI based methods for the assessment of brain age based on magnetic resonance imaging (MRI) scans have recently evolved^5^. The purpose of such data-driven methods is to train models to identify characteristics in the MRI data that are robustly associated with age (or any other key characteristics) in a training set, and then apply the resulting model on a different set of brain scans to estimate the age of individual participants or patients in a clinical context. The discrepancy between the predicted and the chronological age, sometimes referred to as the brain age gap (BAG), can then be used as a proxy of the integrity and health of the individual brain^5^. Most previous brain age studies have pursued feature-based machine learning techniques, which typically require extensive and computationally demanding image processing and feature extraction^5^. However, recent increases in available training data and computational developments have enabled approaches based on deep learning techniques that provide predictions based on minimally processed MRI data. Deep learning techniques may thus enable both more accurate and faster predictions with minimal computational engineering, which facilitates the implementation in a clinical setting^5^. Previous studies have found evidence for higher brain age in AD compared to healthy controls and promising findings for the prediction of disease progression, typically using traditional machine learning^6–8^, but also recently using deep learning^9,10^. A general limitation is that previously performed deep learning studies were not based on patients recruited from everyday clinical practice.

In the present study, we used a recently established deep learning method^11^ to estimate brain age and the corresponding BAG using minimally processed structural MRI brain scans from a heterogeneous memory clinic cohort comprising patients with dementia, mild cognitive impairment (MCI) or subjective cognitive decline (SCD), diagnosed using current clinical criteria. The main purpose was to compare the diagnostic sensitivity and specificity of BAG with conventional brain volumetrics obtained using the clinically available, and previously validated, software NeuroQuant 3. version (NQ, CorTechs labs/University of California, San Diego, CA, USA)^12,13^, using disease stage and etiological diagnosis based on clinical criteria as the gold standard.

## Results

### Sample characteristics

Characteristics of the patients are presented in Table 1. Mean age was 67.1 (SD 9.5) years and 48.7% (113) were females. Of the 232 included patients, 110 had dementia (76 with AD, 6 FTD, 12 DLB, and 16 with other or non-specific dementia) and 122 had non-dementia (45 with SCD and 77 with MCI) with a mean MMSE of 23.5 (SD 5.0) and 28.4 (SD 1.8) respectively, (*p* < 0.001). Patients with dementia were older and had fewer years of education as compared to patients with non-dementia (*p* < 0.001).

**Table 1:** Patient characteristics, and comparisons between disease stages.

	SCD, n 45	MCI, n 77	Dementia, n 110	p*	Non-dementia, n 122	p**
Age, years	61.8 (8.9)	64.6 (9.9)	71.0 (7.7)	** < 0.001**	63.6 (9.6)	** < 0.001**
Female, n (%)	28 (62.2)	31 (40.3)	54 (49.1)	0.064	59 (48.4)	0.912
Education, years, n 212	15.0 (4.1)	14.3 (3.7)	13.3 (3.7)	**0.041**	14.6 (3.9)	**0.019**
MMSE score, n 212	29.1 (1.2)	28.0 (1.9)	23.5 (5.0)	** < 0.001**	28.4 (1.8)	** < 0.001**
CDR-SB, n 222	0.2 (0.3)	1.1 (0.7)	4.8 (2.5)	** < 0.001**	0.8 (0.7)	** < 0.001**
10 word delayed recall, n 176	6.9 (1.6)	4.8 (2.2)	2.0 (2.0)	** < 0.001**	5.5 (2.2)	** < 0.001**
BAG, years	− 1.7 (3.8)	1.4 (4.4)	3.1 (5.3)	** < 0.001**	0.3 (4.4)	** < 0.001**
Forebrain parenchyma/ICV (%)	63.6 (2.6)	62.0 (3.3)	58.6 (3.2)	** < 0.001**	62.6 (3.1)	** < 0.001**
Hippocampi/ICV (%)	0.49 (0.06)	0.46 (0.08)	0.38 (0.07)	** < 0.001**	0.47 (0.07)	** < 0.001**
WMH/ICV (%)	0.23 (0.37)	0.36 (0.42)	0.57 (0.62)	** < 0.001**	0.31 (0.41)	** < 0.001**

All continuous variables expressed as mean (SD).

*Three groups comparison using ANOVA/χ^2^.

**Dementia versus non-dementia (SCD-MCI) comparison using t-test/χ^2^.

*SCD* subjective cognitive decline, *MCI* mild cognitive impairment, *SD* standard deviations, *MMSE* mini mental status examination, *CDR-SB* clinical dementia rating scale sum of boxes, *BAG* brain age gap, *ICV* intracranial volume, *WMH* white matter hypointensities.

Significant values are in bold.

### Brain age prediction

Across groups, age prediction accuracy was high, with a correlation between predicted and chronological age of 0.879 and a mean absolute error (MAE) of 4.29. Within groups the correlation was 0.913 (MAE 3.58) in non-dementia and 0.750 (MAE 5.09) in dementia. Across groups, the correlation between BAG and age was − 0.046 (*p* 0.483); 0.092 (*p* 0.313) in non-dementia and − 0.503 (*p* < 0.001) in dementia.

Diagnostic associations with brain MRI features.

Group-wise summary stats for the MRI features are presented in Table 1. Linear models adjusted for age and sex revealed patients with dementia had higher BAG (t = 5.23, *p* < 0.001), smaller forebrain parenchyma volume (t =  − 6.67, *p* ≤ 0.001), and smaller hippocampi (t =  − 7.36, *p* =  < 0.001) compared to non-dementia patients (unadjusted Cohen’s d of − 0.59, 1.27, and 1.34 respectively).

Table 2 shows the results from the ROC analysis. AUCs were overall higher for the dementia versus SCD classification compared to the dementia versus non-dementia classification. The two NQ measures yielded higher AUCs for both stage classifications compared to BAG, with non-overlapping confidence intervals in the dementia versus non-dementia classification. For the dementia versus SCD classification, the NQ AUCs were both 0.89 (sensitivity 80%, specificity 86–88%) and BAG AUC was 0.78 (sensitivity 80%, specificity 67%). For the dementia versus non-dementia classification, the NQ AUCs were 0.82–0.83 (sensitivity 80%, specificity 67–68%) and BAG AUC was 0.68 (sensitivity 80%, specificity 48%).

**Table 2:** Ability of MRI methods to distinguish dementia from non-dementia (a) and from SCD (b).

	(a) Dementia versus non-dementia			(b) Dementia versus SCD		
	AUC	95% CI	*p*	AUC	95% CI	*p*
BAG	0.678	0.608–0.748	** < 0.001**	0.780	0.703–0.856	** < 0.001**
Forebrain parenchyma/ICV	0.817	0.762–0.872	** < 0.001**	0.893	0.835–0.951	** < 0.001**
Hippocampi/ICV	0.833	0.780–0.886	** < 0.001**	0.890	0.834–0.946	** < 0.001**

*SCD* subjective cognitive decline, *BAG* brain age gap, *ICV* intracranial volume, *AUC* area under the receiver operating curve, *CI* confidence interval.

Significant values are in bold.

Table 3 presents the results from the logistic regression analyses predicting disease stages. The model including hippocampus volume adjusted for demographic covariates gave the highest Nagelkerke R^2^ (0.44 vs. 0.40 and 0.36) for dementia/non-dementia prediction, while the model with BAG adjusted for demographic covariates revealed the highest Nagelkerke R^2^ for dementia/SCD prediction (0.60 vs. 0.58 and 0.59). Adding white matter hypointensity volume (WMH) to model 3 of both diagnostic predictions did not change the Nagelkerke R^2^ substantially (0.36–0.36 and 0.60–0.61) and did not affect the odds ratio or *p* value of BAG. The correlation between forebrain parenchyma and BAG was -0.27 (*p* < 0.001) and between hippocampus volume and BAG it was − 0.22 (*p* < 0.001). In model 4, adding BAG to forebrain parenchyma and covariates, the Nagelkerke R^2^ increased from 0.40 to 0.44, and in model 5, adding BAG to hippocampus and covariates, it increased from 0.44 to 0.48 for dementia/non-dementia prediction (Table 3a). In the dementia/SCD prediction (Table 3b), the Nagelkerke R^2^ increased from 0.59 to 0.69 when adding BAG to forebrain parenchyma and covariates and from 0.58 to 0.68 when adding BAG to hippocampus and covariates.

**Table 3:** Adjusted associations to dementia/non-dementia (a), and to dementia/SCD (b).

	Unadjusted		R^2^*	Adjusted model 1		Adjusted model 2		Adjusted model 3		Adjusted model 4		Adjusted model 5	
	OR (95% CI)	*p*		OR (95% CI)	*p*	OR (95% CI)	*p*	OR (95% CI)	*p*	OR (95% CI)	*p*	OR (95% CI)	*p*
(a)													
Age	1.11 (1.07–1.16)	** < 0.001**	0.23	1.03 (0.98–1.08)	0.237	1.01 (0.96–1.06)	0.707	1.13 (1.08–1.18)	** < 0.001**	1.06 (1.00–1.11)	0.043	1.03 (0.98–1.09)	0.205
Female sex	1.03 (0.60–1.77)	0.909	0.00	1.04 (0.54–2.00)	0.917	1.59 (0.79–3.19)	0.193	1.12 (0.59–2.12)	0.736	0.93 (0.47–1.84)	0.840	1.38 (0.67–2.83)	0.378
Education	0.92 (0.85–0.99)	**0.021**	0.04	0.94 (0.86–1.03)	0.156	0.96 (0.88–1.06)	0.416	0.94 (0.86–1.02)	0.139	0.93 (0.85–1.02)	0.141	0.95 (0.87–1.05)	0.319
Forebrain pc/ICV	0.68 (0.61–0.76)	** < 0.001**	0.38	0.71 (0.63–0.81)	** < 0.001**					0.76 (0.66–0.87)	** < 0.001**		
Hippocampi/ICV	0.5 × 10^−8^ (0.3 × 10^−10^ − 0.1 × 10^–5^)	** < 0.001**	0.43			0.8 × 10^−8^ (0.1 × 10^−10^ − 0.5 × 10^–5^)	** < 0.001**					0.1 × 10^−8^ (1.7 × 10^−10^ − 0.9 × 10^−4^)	** < 0.001**
BAG	1.12 (1.06–1.19)	** < 0.001**	0.10					1.17 (1.09–1.26)	** < 0.001**	1.13 (1.04–1.22)	**0.003**	1.12 (1.04–1.21)	**0.005**
R^2^*					0.40		0.44		0.36		0.44		0.48
(b)													
Age	1.15 (1.09–1.22)	** < 0.001**	0.32	1.06 (0.99–1.13)	0.096	1.02 (0.94–1.09)	0.696	1.20 (1.12–1.29)	** < 0.001**	1.11 (1.02–1.21)	0.013	1.08 (0.99–1.18)	0.068
Female sex	1.84 (0.87–3.88)	0.110	0.03	0.30 (0.10–0.92)	0.034	1.49 (0.51–4.35)	0.470	3.16 (1.04–9.59)	**0.042**	0.22 (0.06–0.81)	0.023	2.16 (0.61–7.65)	0.232
Education	0.89 (0.91–0.98)	**0.021**	0.06	0.96 (0.83–1.10)	0.521	0.98 (0.86–1.12)	0.773	0.94 (0.82–1.08)	0.382	0.95 (0.81–1.12)	0.575	0.95 (0.81–1.11)	0.520
Forebrain pc/ICV	0.55 (0.45–0.67)	** < 0.001**	0.55	0.59 (0.47–0.73)	** < 0.001**					0.66 (0.52–0.84)	** < 0.001**		
Hippocampi/ICV	0.2 × 10^−11^ (0.2 × 10^−15^–0.1 × 10^−7^)	** < 0.001**	0.57			0.1 × 10^−10^ (0.2 × 10^−15^ − 0.4 × 10^−6^)	** < 0.001**					0.9 × 10^−8^ (0.2 × 10^–12^-0.5 × 10^−3^)	**0.001**
BAG	1.22 (1.12–1.34)	** < 0.001**	0.24					1.37 (1.21–1.55)	** < 0.001**	1.33 (1.15–1.54)	** < 0.001**	1.30 (1.13–1.49)	** < 0.002**
R^2^*					0.59		0.58		0.60		0.69		0.68

Logistic regression with dementia (1) versus non-dementia (0) (a), and versus SCD (0) (b), as the dependent variables.

*Nagelkerke R^2^.

*SCD* subjective cognitive decline, *OR* odds ratio, *CI* confidence interval, *Forebrain pc* forebrain parenchyma, *ICV* intracranial volume, *BAG* brain age gap.

Significant values are in bold.

Table 4 summarizes the comparisons of AD, FTD and DLB. BAG and forebrain parenchyma volume, but not hippocampus volume, were significantly different between groups. Post hoc group comparisons of AD versus non-AD, FTD versus non-FTD, and DLB versus non-DLB showed highest BAG in patients with FTD, and largest forebrain parenchyma volume in patients with DLB, compared to the other etiologies (*p* = 0.005 and *p* = 0.012, respectively). AUC of BAG separating FTD from non-FTD was 0.82 (95% CI 0.62–1.00, *p* 0.009) with sensitivity 83% and specificity 82% and of forebrain parenchyma volume separating DLB from non-DLB AUC was 0.73 (95% CI 0.60–0.87, *p* 0.009) with sensitivity 83% and specificity 57%.

**Table 4:** Characteristics of patients with various dementia etiologies, and comparisons of diagnostic groups.

	AD, n 76	FTD, n 6	DLB, n 12	p*	AD versus non-AD	FTD versus non-FTD	DLB versus non-DLB
					p**	p**	p**
Age, years	72.3 (7.5)	64.7 (6.1)	70.2 (8.5)	0.050	**0.048**	**0.023**	0.505
Female, n (%)	41 (53.9%)	4 (66.7%)	2 (16.7%)	**0.039**	0.106	0.399	**0.013**
Education, years (n 84)	12.9 (3.5)	16.4 (3.1)	14.4 (3.4)	0.055	**0.030**	**0.042**	0.285
BAG, years	2.7 (5.2)	8.7 (4.9)	2.6 (3.0)	**0.019**	0.159	**0.005**	0.574
Forebrain parenchyma/ICV (%)	58.2 (3.2)	57.4 (2.2)	60.6 (2.2)	**0.034**	0.117	0.380	**0.012**
Hippocampi/ICV (%)	0.37 (0.07)	0.37 (0.04)	0.40 (0.06)	0.450	0.323	0.893	0.205
WMH/ICV (%)	0.61 (0.58)	0.30 (0.35)	0.53 (0.54)	0.419	0.295	0.215	0.574

*Three group comparison using ANOVA/χ^2^.

**t test.

*AD* Alzheimer’s disease, *FTD* frontotemporal dementia, *DLB* dementia with Lewy bodies, *BAG* brain age gap, *ICV* intracranial volume, *WMH* white matter hypointensities.

Significant values are in bold.

FTD patients were younger than the other patients (64.7 vs. 72.0, *p* = 0.023). When adjusting for age and sex, the association between FTD and BAG was no longer statistically significant (t = 1.74, *p* = 0.086). However, a sensitivity subanalysis including only patients 70 years and below was performed. In this analysis, including five patients with FTD and 34 with non-FTD (median ages 64 and 66 (*p* = 0.117) in FTD and non-FTD respectively), the FTD patients had significantly higher BAG (median 11.3 vs. 5.1 (*p* = 0.004)), and significantly higher BAG when adjusting for age and sex (*p* = 0.007). The AUC of this subgroup analysis was 0.88 (*p* = 0.006).

## Discussion

This study of the diagnostic properties of MRI-based brain age prediction in a memory clinic setting revealed that BAG was associated with disease stage, but the discriminatory power was outperformed by the hippocampus and forebrain parenchyma volumes. BAG was however found to discriminate FTD from other dementia etiologies.

A higher BAG was associated with more impaired disease stage, i.e. dementia versus non-dementia stages. This was as expected as previous studies have found BAG to be associated with cognitive test results and to be higher in AD compared to healthy controls^5^. The association between BAG and disease stage was not confounded by degree of vascular comorbidity, as measured with FreeSurfer WMH. Despite the association with disease stage, BAG performed poorly at discriminating dementia from non-dementia, while volumetrics using NQ did better. We suggest including MCI patients to the non-dementia group might be the cause of the weak discriminating power of BAG as the distinction between MCI and dementia is indefinite and excluding MCI patients should help distinguishing the remaining groups. Thus, in analyses discriminating dementia from SCD, the discriminating power was higher for both MRI measures. Despite increasing the AUC of both methods, BAG did not achieve the sensitivity and specificity levels that are generally expected from a clinical biomarker, while both NQ measures did^14^.

In clinical practice the separation of dementia from non-dementia stages is based on clinical interviews and examinations and does not include biomarkers in the decision-making process. Therefore, the analyses on associations with disease stage were primarily performed to compare the results of the novel, and until now, research-intended brain age prediction with the clinically available hippocampus and forebrain parenchyma volumes. Hippocampal atrophy is a well-known marker of AD^15^ and is often used as a supportive biomarker in the diagnostic workup. It is therefore not surprising that this measure reached clinically relevant discriminatory power as most of the dementia patients had probable AD. The brain age prediction method is trained to capture general brain age and an increased BAG has been associated with genetic, lifestyle, and psychiatric diseases, in addition to AD^7^. Increased BAG is likely to be less specific to neurodegenerative diseases than hippocampal volume, supported by the current findings. It is also possible that cognitive and brain reserve play a greater role when BAG is applied to cognitively impaired patients. We adjusted for educational level, but this only accounts for one part of the complex concept of cognitive reserve^16^. Further, brain age prediction integrating information across the whole brain is likely less sensitive to specific, small region, hippocampal atrophy than hippocampal volume itself. Therefore, it is conceivable that future work performing regional brain age prediction (e.g. Kaufmann et al.^7^) may increase clinical sensitivity and specificity.

NQ volumetrics and BAG were also compared in logistic regression analyses. The adjusted model including hippocampus resulted in a higher Nagelkerke R^2^ than the model with BAG in the dementia/non-dementia prediction, in line with the ROC results. In the dementia/SCD prediction, the adjusted model with BAG had the highest Nagelkerke R^2^. Finally, models including BAG and one of the NQ measures had the highest Nagelkerke R^2^, indicating that NQ volumetrics and BAG provides complementary information for dementia prediction.

The clinical utility of a biomarker ultimately depends on its value for etiological diagnostic work-up. BAG was larger in patients with FTD and the discriminatory power for separating FTD from other etiologies was excellent, with sensitivity and specificity levels above 80%. Previous studies have reported increased brain age in severe mental disorders including schizophrenia, and a genome wide association study found an association between brain ageing and the MAPT gene which encodes for tau protein that is related to FTD^17^. The present sample is relatively small, and the explorative design does not allow for decisive conclusions. Based on previous findings of BAG being associated with diseases associated with frontal lobe pathologies^17,18^ our findings encourage further studies on the association between BAG and FTD and other frontal lobe pathologies. However, the size and distribution of the affected brain regions are expected to influence brain age prediction and could introduce bias to the associations. Indeed, the frontal lobes occupy a relatively large proportion of the brain, accounting for two thirds of the total brain volume^19^. Atrophy of this region could possibly therefore affect brain age estimates to a larger extent than focal atrophy of a smaller brain region, e.g. the hippocampus. Another possibility is that age prediction was biased by age, i.e. that the accuracy of the prediction model varies with age, a phenomenon commonly seen in brain age models^20^. The younger age of the FTD patients may thus have influenced the results. Indeed, when adjusting for age the group differences were attenuated to the point where they no longer reached the threshold for statistical significance. Practically, it is difficult to correct for bias between groups with different age distributions since the true structure of the bias is unknown; correcting jointly in both groups based on independent data can have little to no effect, whereas an in-sample correction could reduce actual group differences. Thus, we performed a sensitivity analysis matching the groups on age, confirming higher BAG in FTD patients.

Hippocampus volume was not significantly smaller in patients with AD dementia compared to non-AD dementia, which might seem unexpected as hippocampal atrophy is known to be a marker of AD. This is however in line with a previous study based on a larger, yet partly overlapping, cohort where hippocampus volume reached an AUC of only 0.62 for discrimination of AD dementia versus non-AD dementia^12^. Further, previous studies from our group concluded that as much as 53% of patients with AD dementia lack atrophy of the hippocampi, and that atypical atrophy patterns are common^21,22^. Both these findings might explain why hippocampus was not able to separate patients with AD dementia from non-AD dementia at a clinically acceptable level, in that study.

There are limitations to the current study. The cross-sectional explorative design and the relatively low number of patients in the etiological comparisons and in the FTD sensitivity analysis limit the confidence and generalizability of the conclusions. Another limitation is that only clinical criteria without AD specific molecular imaging (Aβ-PET) or biofluid (CSF, plasma, Aβ/p-tau) biomarkers were used as the gold standard for the etiological diagnoses. Although the clinical diagnoses were made using the NIA/AA criteria by two experienced physicians, and while the main goal of this study was to examine whether BAG could serve as an additional diagnostic marker in a naturalistic clinical setting, future studies on the diagnostic properties of BAG should include specific biomarkers to substantiate the results. Further, information on comorbidity was not available in the current data set. Although our analyses revealed no substantial confounding effects of white matter cerebrovascular pathology as indexed using WMH from FreeSurfer, various comorbid clinical conditions may influence MRI based analyses and subsequent brain age prediction, and should be considered in future studies.

## Conclusions

Brain age estimation using clinically available MRI scans adds an interesting perspective to the association between brain ageing and neurodegenerative diseases. While NQ volumetrics outperformed BAG in terms of discriminatory power for patients with dementia versus those without dementia, the two measures provided complementary information and we did not find evidence to suggest that our findings were confounded by cerebrovascular comorbidity. The finding of increased brain age in FTD patients is of clinical interest as few biomarkers are available for this diagnosis. The causal direction of effects and prognostic properties remain to be further characterized, preferably in a longitudinal study.

While automated tools for individual-level brain phenotyping based on machine learning, such as brain age prediction, have potential to support clinical diagnostics in a memory clinic setting, further developments and validations of its etiology discriminating and prognostic properties are needed to characterize its clinical potential.

## Methods

### Participants

All patients assessed for cognitive complaints at the memory clinic at Oslo University hospital (OUH), Norway, between June 2015 and January 2019 that met the criteria of SCD, MCI, or dementia (see below), and that had been examined with brain MRI at the same scanner at OUH +/− 6 months from the clinical assessment were eligible for inclusion. Referral to the research MRI scanner at OUH, and not to another MRI scanner, was done at random when an MRI scan was indicated as part of the clinical routine, and it was convenient for the patient due to geography to perform it at OUH. Among the 254 patients fulfilling these criteria, MRI scans of sufficient quality were available from 232 patients, which form the present study cohort.

All patients had consented to be part of a national quality and research register (The Norwegian registry of persons assessed for cognitive symptoms, NorCog). The inclusion and clinical assessments carried out at the memory clinic and data included in NorCog have been described previously^23^.

### Diagnoses and clinical assessments

All patients were diagnosed retrospectively by two experienced physicians (KP and THE), using all available information from the extensive clinical assessments including information from patients and proxies on symptoms, cognitive test results, function in activities of daily living, and physical and psychiatric examinations^23,24^. The NIA/AA 2011 criteria were used to diagnose MCI and dementia^25^, and the Jessen criteria were used to diagnose SCD^26^. Among the patients with dementia, those fulfilling clinical criteria of AD according to NIA/AA 2011 criteria (probable AD and possible AD mixed with vascular pathology)^25^, frontotemporal dementia (FTD) according to the Rascovsky and Gorno-Tempini 2011 criteria^27,28^, and dementia with Lewy bodies (DLB) according to the 2017 McKeith criteria^29^, were included in etiology-based validity analyses. Other diagnoses were excluded due to few cases or other mixed etiologies (i.e., two patients with vascular cognitive impairment, one patient with Parkinson dementia, ten patients with various mixed etiologies, and three unspecific dementia diagnoses). Clinical radiology reports including information on structural pathologies of both cortical and subcortical regions were used to exclude etiologies not related to dementia (i.e. intracranial bleedings or tumors). Further, signs of vascular pathology indicative of cerebrovascular disease and frontal atrophy were used according to criteria of vascular cognitive impairment and FTD. Information on regional structural changes based the clinical report, or from the NQ report was not included in the diagnostic criteria.

The Norwegian version of the Mini Mental State Examination (MMSE) and the Clinical Dementia Rating scale-sum of boxes (CDR-SB) were used as measures of global cognitive and functional performance for descriptive purposes. MMSE gives a score between zero and 30, the higher score the better global cognitive function^30,31^ and the CDR-SB is a global measure of cognitive and functional impairment including six items scored from zero to 3 and summed up to a score ranging from zero to 18, the higher the score the greater the impairment^32^. Two CDR-certified physicians scored the CDR-SB post hoc, based on all available information from the patient records (KP and THE). The Consortium to Establish a Registry for Alzheimer’s Disease (CERAD) 10-word delayed recall test, with scores from zero to 10, the higher score the better the learning and retrieving capacity^33^ was included as a descriptive measure of memory function.

### MRI acquisition and analysis

All patients were assessed with brain MRI according to the same research protocol using a GE Discovery MR750 3T scanner (GE Healthcare, Milwaukee, WI, US). Whole brain T1-weighted structural MRI data was acquired using an inversion recovery‐fast spoiled gradient echo sequence (BRAVO) with the following parameters: TR = 8.16 ms, TE = 3.18 ms, TI = 450 ms, flip angle = 12°, field of view = 256 mm, acquisition matrices = 256 × 256, 188 sagittal slices, slice thickness = 1.0 mm, voxel size = 1 × 1 × 1 mm^3^. The scans were analyzed following a previously established minimal processing pipeline and brain age prediction model^11^, and with NeuroQuant 3. version (NQ, CorTechs labs/University of California, San Diego, CA, USA)^13^.

Brain age was computed using a state-of-the-art deep convolutional neural network, trained on a large population dataset (N = 53,542 from 21 publicly accessible datasets) with a wide age range from a multitude of scanners^11^, not including the one used for the present study. The model is available online^34^. As input, the model used minimally processed imaging data linearly registered with six degrees of freedom to MNI152 space. BAG was calculated by subtracting chronological age from predicted brain age, such that a positive BAG reflects higher predicted age compared to chronological age and vice versa.

NQ produces valid and reliable volumetric measures of several brain regions^13,35^. NQ volumetry of hippocampus correlates well with visual ratings of the medial temporal lobe using the Scheltens scale^36^. In the present study, we included the volume of the hippocampus as atrophy of the hippocampus is one of the best-established diagnostic imaging biomarkers for AD^15^, constituting the majority of the patients with dementia in the present sample. Additionally, we included forebrain parenchyma volume, including all parenchymal brain volumes except the brainstem and cerebellum, as this volume was previously shown to have the best ability to discriminate between dementia and non-dementia^12^, and to include a measure that would represent more than the AD specific medial temporal region. Both structures were included as proportions of estimated intracranial volume, i.e. the sum of whole brain volume and CSF spaces, to adjust for head size.

In 229 of the 232 included patients, FreeSurfer data on white matter hypointensities (WMH) was available^37^. Although based on T1-weighted MRI scans, this measure has been found to correlate well with both state-of-the-art T2/FLAIR white matter hyperintensities and the visual rating scale of Fazekas^38–40^. To adjust for head size, WMH was divided by the FreeSurfer measure of total intracranial volume. WMH was included post hoc to evaluate if cerebrovascular comorbidity could confound the association between BAG and dementia.

### Statistics

Data were analyzed using IBM SPSS Statistics for Windows (version 27, Armonk, NY, USA). The significance level was set at 0.05. Diagnostic groups were compared using independent samples t-test and ANOVA for continuous measures and χ^2^ tests for categorical measures. Age- and sex-adjusted linear models were performed for group-wise comparisons of the MRI measures. Medians and Mann–Whitney U test were used in a sensitivity analysis of the subgroup of patients 70 years of age and below.

To compare the validity of the two MRI methods, receiver operating characteristics (ROC) analyses were carried out for each method, calculating the area under the curve (AUC) as a measure of the performance of the classifiers to separate dementia from non-dementia and from SCD. The interpretation of the AUC depends on the clinical setting in which the test should be used, but generally an AUC of 0.5–0.7 is regarded poor, 0.7–0.8 acceptable, 0.8–0.9 excellent, > 0.9 outstanding^41^. For a biomarker to be clinically useful, the sensitivity and specificity should be at least 80%^14^. Thus, for each MRI measure, the sensitivity was set at 80% and the specificity was obtained from the ROC analysis.

Bivariate Pearson correlation of the three MRI measures (BAG, hippocampus volume and forebrain parenchyma volume) was performed to prepare the logistic regression analysis. Hippocampus volume and forebrain parenchyma volume were highly correlated (r = 0.703, *p* < 0.001) Thus, in the logistic regression analyses predicting dementia versus non-dementia and dementia versus SCD, the forebrain parenchyma, hippocampus volume, and BAG were included in separate models (models 1, 2, and 3), adjusting for demographic covariates (age, sex, and educational level). The Nagelkerke R^2^ was used as an estimate of the explained variance to compare the models. In models 4 and 5, BAG was added to each of the two volumetric measures to assess its additive value for the prediction of disease stage. Finally, to explore if cerebrovascular comorbidity could confound the association between BAG and diagnosis, WMH was added to model 3 (not in table).

### Ethics declarations

All patients gave written informed consent to be included in NorCog. The Regional Committee of Medical Research Ethics of the South-East Norway approved the use of NorCog data in the present study (REC South-East number 29461). All methods and analyses were performed in accordance with the Declaration of Helsinki.

## Data Availability

Data from this study are not made publicly available. The regional ethics committee has not approved data delivery outside of Europe, consent for publication of raw data was not obtained from the participants of the study, and complete anonymization is difficult to achieve. Data are available from the National Advisory Unit on Ageing and Health for researchers who meet the criteria for access to confidential data. Data requests can be addressed to post@aldringoghelse.no.
